# Potential impact and cost-effectiveness of future ETEC and *Shigella* vaccines in 79 low- and lower middle-income countries

**DOI:** 10.1016/j.jvacx.2019.100024

**Published:** 2019-04-18

**Authors:** John D. Anderson, Karoun H. Bagamian, Farzana Muhib, Ranju Baral, Lindsey A. Laytner, Mirna Amaya, Thomas Wierzba, Richard Rheingans

**Affiliations:** a Goodnight Family Department of Sustainable Development, Appalachian State University, 222 Living Learning Center, 305 Bodenheimer Drive, Boone, NC 28608, USA; b Emerging Pathogens Institute, P.O. Box 100009, 2055 Mowry Road, Gainesville, FL 32610, USA; c Department of Environmental and Global Health, University of Florida, Gainesville, FL 32603, USA; d Bagamian Scientific Consulting, 978 SW 2^nd^Ave., Gainesville, FL 32601, USA; e PATH, 455 Massachusetts Ave. NW, Suite 1000, Washington, DC 20001, USA; f PATH, 201 Westlake Avenue, Suite 200, Seattle, WA 98121, USA

**Keywords:** ETEC, *Shigella*, Diarrhea, Stunting, Cost-effectiveness, Vaccines

## Abstract

While diarrhea mortality in children has declined over the last two decades, there has been a slower decline in diarrheal episodes. Repeated diarrheal episodes are associated with childhood stunting, which leads to increased mortality risk from infectious diseases. Vaccine candidates are under development for enterotoxigenic *Escherichia coli* [ETEC] and *Shigella*, important enteric pathogens in children in low income countries. These future vaccines could significantly reduce diarrheal burden, prevent ETEC- and *Shigella*-induced stunting, and stunting-associated mortality.

We developed a cost-effectiveness model for two putative standalone ETEC and *Shigella* vaccine candidates to evaluate vaccine impact on mortality, morbidity, stunting, and stunting-associated deaths from other infectious diseases. We modeled impact over the first ten years after vaccine introduction in children under five years old living in 79 low and low-middle income countries.

ETEC and *Shigella* diarrhea would cause an estimated 239,300 [95% UL: 179,700–309,800] and 340,300 [256,500–440,800] child deaths, respectively, from years 2025 to 2034. Most of these deaths would occur in AFRO countries. ETEC and *Shigella* moderate-to-severe diarrheal episodes would result in over 13.7 [8.4–19.0] and 21.4 [13.1–29.8] million stunted children, respectively. Introducing ETEC or *Shigella* vaccine each with 60% efficacy could prevent 92,000 [61,000–129,000] ETEC and 126,600 [84,000–179,000] *Shigella* direct deaths and 21,400 [11,300–34,800] ETEC- and 34,200 [18,000–56,000] *Shigella*-induced stunting deaths. ETEC ICERs ranged from $2172/DALY [1457–4369] in AFRO to $19,172/DALY [12,665–39,503] in EURO. *Shigella* ICERs ranged from $952/DALY [632–2001] in EMRO to $640,316/DALY [434,311–1,297,192] in EURO.

Limitations of this analysis include uncertainty of vaccine efficacy, duration of protection, and vaccine price. Inclusion of other infectious disease mortality due to stunting provides a more accurate assessment of total ETEC and *Shigella* disease burden and increased the projected impact and cost-effectiveness of vaccination. Introducing vaccines only in high burden countries and regions could substantially reduce cost without substantially reducing impact.

## 1 Introduction

Globally, diarrhea remains the second leading cause of mortality, accounting for approximately 500,000 deaths annually in children under five years old [1]. However, diarrheal mortality in children has declined by 34.3%, with similar declines in Shigellosis (33.8%) and enterotoxigenic *E. coli* (ETEC) infection (38.1%) from 2005 to 2015 [2]. While diarrheal mortality has declined, morbidity and mortality continue to plague many low- and lower middle-income countries (LMICs).

In addition to rotavirus, other pathogens have a substantial role in diarrheal burden. The Global Enteric Multicenter Study (GEMS) found that of 22 diarrheal pathogens, four—rotavirus, *Shigella*, ETEC, and *Cryptosporidium*—were associated with moderate-to-severe diarrhea (MSD), accounting for 70% of cases in 0–4 year olds [3]. This study also documented an increased mortality risk for MSD ETEC cases and increased stunting risk in cases associated with both ETEC and *Shigella* [4]. ETEC and *Shigella* have been among the top four causes of diarrhea associated with years lost to disability (YLDs) worldwide [5]. A recent study evaluating global ETEC and *Shigella* burden found that these pathogens have a sizeable global burden, especially in the World Health Organization (WHO) designated African and the Eastern Mediterranean regions [6].

As diarrheal mortality has declined, there is increased focus on diarrhea morbidity in pediatric populations. Repeated, non-fatal episodes of diarrhea from infection by certain pathogens are thought responsible for reduced linear growth and childhood stunting [7], [8], [9], increasing mortality risk from other infectious diseases [10]. Results from GEMS showed that MSD episodes shifted the height-for-age z-scores downward [3], [11], increasing a child’s risk of stunting. In countries where stunting is highly prevalent, ETEC- and *Shigella*-induced stunting impacts even more children [12].

The burden from these pathogens necessitates new prevention strategies. A potential and highly beneficial prevention strategy would be the use of vaccines to prevent ETEC and *Shigella* infection, which are currently under development with Phase 1 and Phase 2 data available shortly in non-infant populations. In addition to reducing burden worldwide, the successful rollout of rotavirus vaccines has shown that enteric vaccines are deployable to endemic countries. Studies describing rotavirus burden, vaccine impact, and cost-effectiveness have contributed to global and country decisions to accelerate rotavirus vaccine introduction [13], [14]. Therefore, investigating how ETEC and *Shigella* vaccination could impact high burden countries or populations is not only important in guiding vaccination programs, but may in turn spur action by policymakers.

In this study, we conducted a vaccine impact and cost-effectiveness analysis for ETEC and *Shigella* vaccines to identify high-need areas and to capture the full potential value of these vaccines. We evaluated the impact of vaccination on the mortality, morbidity, number of stunted children, and stunting-associated deaths from other infectious diseases, to understand the expanded impact of these vaccines.

## 2 Materials and methods

### 2.1 Population and time frame

We included seventy-nine countries from a previous analysis of ETEC and *Shigella* burden with a full description of country inclusion and exclusion criteria [6] (Table 1). We aggregated national estimates by WHO regions to identify trends (‘AFRO’: African region, ‘AMRO’: Region of the Americas, ‘EMRO’: Eastern Mediterranean Region, ‘SEARO’: Southeast Asian Region, ‘WPRO’: Western Pacific Region). We assumed all countries would introduce the vaccines in 2025. We examined 10 annual birth cohorts of children over the first five years of life. Population estimates of under five children were based on UN Population Division country estimates and projections from 2025 to 2034 [15].

**Table 1:** The 79 Countries included in study by WHO region. *AFRO: African region, AMRO: Region of the Americas, EMRO: Eastern Mediterranean Region, SEARO: Southeast Asian Region, WPRO: Western Pacific Region.*

AFRO		AMRO	EMRO	EURO	SEARO	WPRO	Excluded
Angola	Liberia	Bolivia	Afghanistan	Armenia	Bangladesh	Cambodia	Cabo Verde
Benin	Madagascar	El Salvador	Djibouti	Georgia	Bhutan	Kiribati	Kosovo
Burkina Faso	Malawi	Guatemala	Egypt	Kyrgyz Republic	DPR Korea	Lao PDR	Micronesia
Burundi	Mali	Haiti	Jordan	Moldova	India	Mongolia	Moldova
Cameroon	Mauritania	Honduras	Morocco	Tajikistan	Indonesia	Papua New Guinea	Vanuatu
Central African Republic	Mozambique	Nicaragua	Pakistan	Ukraine	Myanmar	Philippines	
Chad	Niger		Somalia	Uzbekistan	Nepal	Solomon Islands	
Comoros	Nigeria		Sudan		Sri Lanka	Viet Nam	
Congo	Rwanda		Syria		Timor-Leste		
Congo DR	São Tomé & Principe		Tunisia				
Côte d’Ivoire	Senegal		Yemen				
Eritrea	Sierra Leone						
Ethiopia	South Sudan						
The Gambia	Swaziland						
Ghana	Tanzania						
Guinea	Togo						
Guinea-Bissau	Uganda						
Kenya	Zambia						
Lesotho	Zimbabwe						

### 2.2 Burden of diarrhea mortality and morbidity

#### 2.2.1 Etiological fraction

ETEC and Shigella burden is dependent upon the fraction of episodes and deaths attributable to each [3], [16]. As previously described [6], we used culture-based etiological estimates [3] and adjusted them for under-detection using estimates derived from molecular methods based on Liu et al. [17], [18]. We applied an adjustment of 1.5 times for ETEC and 2.0 times for *Shigella* to the culture results [20].

#### 2.2.2 Diarrhea mortality and morbidity estimates

Similar to our burden study, we used the mid-point of 2015 diarrheal mortality estimates from two sources: the Global Burden of Disease study (GBD) at the Institute for Health Metrics and Evaluation (IHME) [19] and the WHO Maternal Child Epidemiology Estimation (MCEE) project [20]. We then used the etiological fraction for ETEC and *Shigella* to calculate pathogen-attributable deaths and adjusted diarrheal mortality to account for countries that introduced rotavirus vaccine before 2014 [6]. We projected mortality and morbidity estimates from 2015 to 2034. Because diarrheal mortality rates have declined over time, we estimated annual rates of decline for non-rotavirus under-five diarrheal mortality in each country using data from Child Health Epidemiology Reference Group from 2000 to 2013 [21]. We calculated diarrhea morbidity estimates using WHO region-specific estimates of diarrhea episodes and the etiological fractions for ETEC and *Shigella* [22]. We assumed morbidity declined at a rate of 0.45% per year. This decline percentage was calculated from YLDs from diarrheal disease from 1990 to 2010 from the GBD [23].

#### 2.2.3 Effects of ETEC- and Shigella-induced stunting

We applied the methods from Anderson et al. [6] to determine the effects of ETEC and Shigell-induced stunting. First, we calculated the shift in child height-for-age z-scores from ETEC and *Shigella* episodes using GEMS results (Table 1). In the absence of reliable community-level estimates for diarrhea treatment in countries included in this study, we assumed 22% of diarrheal episodes where care was sought at a health facility were considered MSD. The proportion of child diarrheal episodes where caretakers sought care were taken from the most recent Demographic and Health Surveys (DHS), available for 70 countries [24]. In countries without a country-level estimate, we substituted the corresponding WHO regional average. Based on the mean of estimates for the countries included in this analysis, we calculated that 47% of caretakers would visit a health facility after the child experienced an episode of diarrhea (Table 1). Thus, we assume that 10% (22% × 47%) of child diarrheal episodes were moderate-to-severe in each region. We used the same the approach as in Anderson et al. [6] to estimate the number of child deaths from infections for which stunting is a risk factor (pneumonia, malaria, measles, and diarrhea [25]). We did not project rate of change of pneumonia, malaria and measles burden over time.

### 2.3 Outcomes measures

Outcome measures included: diarrheal episodes; direct deaths; children stunted; stunted children dying from other infectious diseases; and Disability-adjusted Life Years (DALYs). Stunting is a risk factor for other infectious disease deaths and not directly included in DALY calculations. All mortality outcomes, including other infectious disease deaths due to induced stunting, were translated to DALYs using standard techniques [26], [27]. We calculated DALYs using non-uniform age-weighting and a 3% annual discount rate. We calculated all outcomes annually and cumulatively from time of introduction.

### 2.4 Vaccines

We evaluated the impact of potential ETEC and a *Shigella* standalone vaccine candidates when introduced nationally in 79 countries. We assume each vaccine is 60% efficacious in preventing deaths and MSD episode. Our other assumptions were that protection was conferred after the third dose and that there was no protection for partially vaccinated children. We assumed that vaccine effectiveness does not wane, but we also assumed that there was no effectiveness after five years of age or herd protection. For coverage, we used country specific 2015 DPT3 coverage estimates [28] and only included estimates for children estimated to receive a full course (all three doses).

As vaccine price is uncertain, we used the Gavi Rotarix price of approximately $2.00 per dose [29] as our basis. For our study, we assumed study vaccines would cost $3.30 per dose, and we varied this price in the sensitivity analysis to assess price impact on cost-effectiveness.

### 2.5 Costs

As there are no published medical costs for ETEC and *Shigella* diarrhea for the 79 study countries, we used country specific estimates of direct medical costs of illness associated with inpatient and outpatient care for MSD episodes. We assumed 1 of 8 outpatients with MSD were referred for inpatient care [13], [30].

Direct medical costs were based on WHO-CHOICE Service Delivery Unit Cost estimates and commodity costs. For outpatient medical costs, we used country specific cost per outpatient visit at a primary hospital. We used country specific daily costs at hospitals and a four-day stay to calculate inpatient cost. We assumed that outpatients receive six oral rehydration solution packets per day for two days, and inpatients receive six packets per day and two IVs during a four day hospital stay [31]. On average, the illness cost per episode was $10.05 for outpatients and $82.25 for inpatients. These estimates were triangulated against country specific estimates, when available in the literature [32], [33], [34], [35], [36], [37], [38], [39], [40], [41], [42], [43], [44], [45], [46], [47]. In most cases, modelled estimates were aligned with empirical estimates.

Using data available from Portnoy et al. [48], we generated vaccine administration costs. All costs were in 2016 US dollars and discounted (3%). Our cost-effectiveness estimates were from the health system perspective.

### 2.6 Cost-effectiveness

We calculated vaccination cost (V) cumulated over the first 10 years (t) after introduction starting in 2025 for each country (c) based on vaccine administration cost, vaccine price, and quantity (birth cohort times coverage rate with 10% vaccine wastage). We calculated averted costs (A) based on population, vaccine coverage, efficacy, and access to care and medical costs in each country (c). We calculated net costs (N) for each region (r) as:

$$N_{r}=\sum_{t=10}^{r}\left(V_{c}-A_{c}\right)$$

We calculated vaccine benefits (B) for each region (r) based on the sum of population, coverage (C), efficacy (E), and DALY burden (D) in each country (c) cumulated over the first 10 years (t) after introduction. We calculated the number of children fully vaccinated each year by multiplying the annual birth cohort by the assumed vaccine coverage. We projected benefits for the first five years of life for children vaccinated in each annual birth cohort.

$$B_{r}=\sum_{t=10}^{r}{(C}_{c}\cdot E_{c}\cdot D_{c}$$

Our primary cost-effectiveness measure was the regional Incremental Cost-Effectiveness Ratio (${ICER}_{r}$), which is the aggregated country-level incremental costs associated with introducing each vaccine divided by aggregated country-level health benefit.

$${ICER}_{r}=\frac{\sum_{t=10}^{r}N_{r}}{{\sum_{t=10}^{r}B}_{r}}$$

Our comparator scenario was no vaccination. We calculated ICERS with and without other infectious disease burden due to stunting for each country and region annually and cumulatively.

We presented results using two thresholds for cost-effectiveness as compared to GDP [49]. We presented results for countries where ICERS are less than 3 times their GDP, which are considered cost effective and results for countries where ICERS are less than GDP, which has historically been used to determine if an intervention is ‘highly’ cost-effective.

### 2.7 Sensitivity analysis

In order to assess the impact of uncertainty and changes in key input variables, we conducted two types of sensitivity analysis using SimVoi [50]. First, we used one-way sensitivity analysis to demonstrate the impact of individual input parameters on vaccination cost-effectiveness. These results are shown in tornado diagrams, with each horizontal band showing the effect of varying each parameter between high and low values. Second, we conducted a probabilistic sensitivity analysis (PSA) to show the overall impact of input parameter uncertainty (Table 2) on our estimates of key outcomes. Monte Carlo analysis using 10,000 iterations was conducted and we included estimated upper and lower 95% uncertainty limits (2.5% and 97.5%) for key outputs in brackets after our estimates. We reported results of the sensitivity analyses as a range of the difference between high and low ICER estimates.

**Table 2:** Model parameters for base case scenario and ranges used in uncertainty and sensitivity analyses.

Model input	Values	Range	Reference
*Burden*			
Population estimates	Varies by country	–	[17]
Diarrheal mortality for children under 5 years of age	Varies by country	±10%; Triangular	[1], [22], [25]
Change in non-rotavirus under-5 diarrheal mortality rate	Varies by country; mean = 12.8% decline	±25%; Triangular	[23]
Diarrheal episodes in children under 5 years of age	Varies by region; 2.2–3.3 episodes/child annually	–	[6], [56]
Etiological fraction attributed to ETEC by WHO region	Varies by WHO region; 0.075–0.123	±25%; Triangular	[20], [24]
Etiological fraction attributed to Shigella by WHO region	Varies by WHO region; 0.002–0.238	±25%; Triangular	[20], [24]
Stunting induced by ETEC episodes	0.068 shift in HAZ	±50%; Triangular	[3], [6]
Stunting induced by *Shigella* episodes	0.082 shift in HAZ	±50%; Triangular	[3], [6]
Fraction of diarrhoeal episodes that are moderate to severe	22% of children who sought care at a healthcare facility	0.12–0.32; Triangular	[3], [6]
Percentage of caretakers that sought care at a health facility after a child’s diarrheal episode	47%	44–58%, Not varied in uncertainty analysis	[24]

*Vaccination and Medical Costs*			
ETEC vaccine efficacy	60%	±20%; Triangular	Assumption
*Shigella* vaccine efficacy	60%	±20%; Triangular	Assumption
Dose price	$3.30	$2.50–$7.00; Triangular	Assumption
Administration cost	LI = $1.93, LMI = $1.64	±40%; Triangular	[48]
Cost of ETEC or *Shigella* illness	Varies by country; outpatient mean = $6.09/episode; inpatient mean = $51.07/episode	±40%; Triangular	[32], [33], [34], [35], [36], [37], [38], [39], [40], [41], [42], [43], [44], [45], [46], [47]
Inpatient visit rate	12.5% of outpatient visits	±50%; Triangular	[30]
Outpatient visit rate (cases taken to healthcare facility)	Varies by country; Mean of 47% ETEC or *Shigella* cases sought treatment annually	44–58%, Not varied in uncertainty analysis	[24]

## 3 Results

### 3.1 Expected outcomes over time

Over 10-years in 79 LMICs, ETEC and *Shigella* would cause an estimated 239,300 [95% CI: 180,100; 310,000] and 340,300 [256,700; 440,800] deaths, respectively, in children under five without vaccination (Table 3, Table 4). Most deaths would occur in AFRO for ETEC (68%) and *Shigella* (54%). In addition, MSD episodes of ETEC and *Shigella* would result in 13.7 [8.3; 19.1] and 21.4 [13.2; 29.8] million stunted children, respectively. These cases of stunting result in an additional 45,400 [28,700; 59,800] and 72,900 [47,600; 91,900] deaths due to other infectious diseases indirectly attributable to ETEC and *Shigella*, respectively. The global burden of ETEC and *Shigella* in LMICs is estimated at 5.3 [4.0; 6.8] and 7.5 [5.6; 9.7] deaths/100,000 children, respectively, with the highest rates in EMRO (*Shigella*) and AFRO (ETEC) regions. Globally, ETEC would cause 93 [90; 96] million MSD episodes while *Shigella* would cause 118 [115; 121] million MSD episodes (Table 2, Table 3). AFRO accounts for 49% and 43% of global ETEC and *Shigella* episodes, respectively, followed by SEARO at 28% (ETEC) and EMRO at 32% (*Shigella*).

**Table 3:** Estimated disease burden associated with ETEC infection and ETEC vaccination impact in children under 5 years of age in 79 countries, by region. Model estimates are projected from 2025 (year of introduction) to 2034. Results from uncertainty analysis are listed below model estimates. Upper and Lower represent 95% uncertainty intervals for each estimate.

	AFRO	AMRO	EMRO	EURO	SEARO	WPRO	GAVI-eligible	Global
Number of countries	38	6	11	7	9	8	48	79
*ETEC disease burden*								
MSD episodes (millions)	45.81	2.25	15.00	1.15	25.64	3.00	49.59	92.84
	[44.57; 47.05]	[2.19; 2.31]	[14.31; 15.68]	[1.13; 1.16]	[24.90; 26.38]	[2.95; 3.04]	[48.00; 51.19]	[90.05; 95.64]
ETEC-induced stunting cases (millions)	6.53	0.28	2.01	0.15	4.33	0.38	7.08	13.68
	[3.99; 9.08]	[0.17; 0.40]	[1.22; 2.80]	[0.09; 0.21]	[2.64; 6.02]	[0.23; 0.53]	[4.32; 9.85]	[8.35; 19.02]
Total deaths* (1000 s)	161.92	2.37	34.98	0.48	36.85	2.65	135.62	239.25
	[121.88; 209.32]	[1.76; 3.09]	[26.51; 44.78]	[0.35; 0.64]	[26.96; 48.88]	[2.00; 3.41]	[102.26; 175.17]	[179.73; 309.81]
Total deaths*/100,000 children**	8.56	3.68	5.54	0.70	2.20	1.18	6.34	5.25
	[6.44; 11.07]	[2.74; 4.80]	[4.20; 7.09]	[0.51; 0.93]	[1.61; 2.92]	[0.888; 1.510]	[4.78; 8.19]	[3.94; 6.80]
Other Infectious disease deaths from ETEC-induced stunting as a percentage of total ETEC deaths	20	13	19	14	16	14	19	19
	[13; 26]	[8; 17]	[12; 24]	[9; 18]	[10; 21]	[9; 18]	[13; 25]	[12; 25]
Total DALYs* (1000 s)	5613.1	85.3	1220.9	18.6	1302.7	97.5	4720.2	8338.1
	[4226.8; 7252.5]	[63.4; 111.3]	[925.3; 1562.5]	[13.6; 24.8]	[953.0; 1728.4]	[73.7; 125.4]	[3559.8; 6093.1]	[6267.0; 10,798.9]
Total DALYS* /100,000 children**	296.7	132.6	193.4	27.2	77.8	43.2	220.7	183.0
	[223.4; 383.4]	[98.6; 173.1]	[146.6; 247.5]	[19.8; 36.1]	[56.9; 103.2]	[32.7; 55.6]	[166.4; 284.9]	[137.6; 237.0]
Outpatient treatment costs (millions US$)	190.8	12.5	77.1	7.7	174.8	17.4	215.2	480.1
	[131.7; 250.7]	[8.6; 16.4]	[53.2; 101.3]	[5.3; 10.1]	[120.7; 229.6]	[12.0; 22.8]	[148.6; 282.7]	[331.5; 630.9]
Inpatient treatment costs (millions US$)	160.8	15.1	76.6	10.6	207.6	20.0	161.7	490.6
	[87.6; 252.8]	[8.2; 23.7]	[41.7; 120.3]	[5.8; 16.7]	[113.0; 326.2]	[10.9; 31.5]	[88.1; 254.2]	[267.2; 771.2]

*ETEC vaccination IMPACT*								
MSD episodes averted (millions)	22.61	1.21	7.10	0.57	13.77	1.72	25.45	46.97
	[16.76; 28.46]	[0.89; 1.52]	[5.26; 8.94]	[0.43; 0.72]	[10.21; 17.34]	[1.27; 2.16]	[18.86; 32.04]	[34.82; 59.14]
ETEC-induced stunting cases averted (millions)	3.17	0.15	0.92	0.07	2.32	0.22	3.57	6.85
	[1.81; 4.79]	[0.09; 0.23]	[0.53; 1.39]	[0.04; 0.10]	[1.3; 3.5]	[0.12; 0.33]	[2.03; 5.38]	[3.91; 10.34]
Direct diarrheal deaths averted (1000 s)	59.31	1.01	13.44	0.24	16.73	1.24	53.72	91.97
	[39.86; 83.85]	[0.67; 1.45]	[9.06; 18.89]	[0.16; 0.35]	[11.00; 24.17]	[0.83; 1.75]	[36.11; 75.97]	[61.55; 130.39]
Direct diarrheal deaths averted/100,000 FVC***	3.80	1.75	2.47	0.44	1.10	0.60	2.85	2.33
	[2.55; 5.37]	[1.16; 2.50]	[1.66; 3.47]	[0.29; 0.64]	[0.72; 1.59]	[0.40; 0.85]	[1.92; 4.04]	[1.56; 3.31]
Infectious disease deaths due to induced stunting averted (1000 s)	14.68	0.15	3.09	0.04	3.21	0.20	12.99	21.36
	[7.89; 23.90]	[0.08; 0.25]	[1.66; 5.00]	[0.02; 0.06]	[1.70; 5.29]	[0.11; 0.32]	[6.98; 21.15]	[11.47; 34.81]
Infectious disease deaths due to induced stunting/100,000 FVC	0.94	0.26	0.57	0.07	0.21	0.10	0.69	0.54
	[0.50; 1.53]	[0.14; 0.43]	[0.31; 0.92]	[0.04; 0.11]	[0.11; 0.35]	[0.05; 0.15]	[0.37; 1.12]	[0.29; 0.88]
Total deaths* averted (1000 s)	73.99	1.16	16.53	0.28	19.94	1.43	66.72	113.33
	[49.43; 105.71]	[0.77; 1.67]	[11.08; 23.48]	[0.18; 0.41]	[13.07; 29.10]	[0.96; 2.04]	[44.59; 95.28]	[75.49; 162.29]
Total deaths* averted /100,000 FVC***	4.74	2.01	3.04	0.51	1.31	0.69	3.54	2.87
	[3.16; 6.77]	[1.33; 2.89]	[2.04; 4.31]	[0.33; 0.75]	[0.86; 1.92]	[0.463; 0.984]	[2.37; 5.06]	[1.91; 4.11]
Total DALYs* averted (1000 s)	2532	40	563	9	683	49	2281	3876
	[1690; 3617]	[26; 57]	[378; 800]	[6; 14]	[448; 997]	[33; 69]	[1524; 3259]	[2580; 5549]
Total DALYS* averted/100,000 FVC***	162	69	104	17	45	24	121	98
	[108; 232]	[46; 99]	[69; 147]	[11; 26]	[29; 66]	[16; 34]	[81; 173]	[65; 141]
Vaccination costs (millions US$)	5685	206	1928	191	5365	730	6835	14,104
	[4501; 9204]	[163; 336]	[1523; 3150]	[151; 313]	[4237; 8774]	[576; 1194]	[5412; 11,075]	[11,149; 22,976]
Vaccination costs/100,000 FVC***	363,978	355,339	354,231	352,802	353,201	352,645	363,155	357,608
	[288,159; 589,232]	[280,801; 579,870]	[279,885; 578,803]	[278,605; 577,225]	[278,989; 577,647]	[278,455; 577,074]	[287,524; 588,425]	[282,681; 582,536]
Administration costs/100,000 FVC***	105,908	98,125	97,127	95,840	96,199	95,698	105,166	100,169
	[72,792; 138,495]	[67,442; 128,317]	[66,756; 127,012]	[65,872; 125,329]	[66,118; 125,799]	[65,774; 125,144]	[72,282; 137,525]	[68,847; 130,991]
Medical costs averted (millions US$)	173.8	15.3	80.5	7.5	207.7	20.5	199.8	505.3
	[104.8; 263.3]	[9.0; 23.5]	[48.1; 122.7]	[4.4; 11.6]	[122.8; 319.3]	[12.2; 31.5]	[120.9; 301.7]	[301.7; 771.6]
Net costs (millions US$)	5511	191	1847	184	5157	709	6635	13,599
	[4328; 9028]	[147; 321]	[1441; 3067]	[143; 305]	[4024; 8558]	[556; 1173]	[5213; 10,871]	[10,641; 22,453]
ICER without stunting burden (2016 US$ / DALY)	2726	5496	4027	22,266	9019	16,719	3621	4334
	[1839; 5367]	[3616; 11,202]	[2712; 8012]	[14,678; 45,193]	[5972; 18,218]	[11,274; 33,246]	[2446; 7130]	[2912; 8625]
ICER with stunting burden (2016 US$ / DALY)	2177	4777	3279	19,434	7549	14,540	2909	3508
	[1469; 4334]	[3140; 9797]	[2214; 6591]	[12,789; 39,598]	[4983; 15,387]	[9,811; 29,020]	[1964; 5788]	[2357; 7037]

NOTE: Though vaccinations occur annually from 2025 to 2034, impacts are projected over the first five years of the vaccinated child’s life. Thus, the last year included in impact estimates is 2039.

* Total deaths and DALYS are the sum of ETEC burden attributed to diarrhea from ETEC infection (direct) and ETEC-induced deaths from other infectious diseases.

** Children: children under 5 years of age.

*** Fully vaccinated children (FVC): Number of eligible children who received all three doses of the vaccine.

**Table 4:** Estimated disease burden associated with *Shigella* infection and *Shigella* vaccination impact in children under 5 years of age in 79 countries, by region. Model estimates are projected from 2025 (year of introduction) to 2034. Results from uncertainty analysis are listed below model estimates. Upper and Lower represent 95% uncertainty intervals for each estimate.

	AFRO	AMRO	EMRO	EURO	SEARO	WPRO	GAVI-eligible	Global
Number of countries	38	6	11	7	9	8	48	79
*Shigella disease burden*								
MSD episodes (millions)	50.61	2.31	37.21	0.09	27.59	0.36	68.82	118.16
	[49.38; 51.85]	[2.24; 2.37]	[36.53; 37.90]	[0.08; 0.11]	[26.85; 28.33]	[0.31; 0.40]	[67.25; 70.42]	[115.39; 120.96]
*Shigella*-induced stunting cases (millions)	9.02	0.35	6.38	0.01	5.60	0.05	12.58	21.41
	[5.53; 12.54]	[0.21; 0.49]	[3.91; 8.86]	[0.01; 0.02]	[3.44; 7.78]	[0.03; 0.08]	[7.71; 17.50]	[13.12; 29.77]
Total *Shigella* deaths* (1000 s)	183.79	2.49	112.59	0.02	41.26	0.17	224.34	340.32
	[138.51; 238.18]	[1.87; 3.26]	[85.71; 144.32]	[0.01; 0.02]	[30.44; 54.88]	[0.13; 0.22]	[169.92; 289.29]	[256.54; 440.83]
Total *Shigella* deaths*/100,000 children**	9.72	3.88	17.83	0.02	2.46	0.08	10.49	7.47
	[7.32; 12.59]	[2.90; 5.06]	[13.58; 22.86]	[0.02; 0.03]	[1.82; 3.28]	[0.057; 0.097]	[7.94; 13.53]	[5.63; 9.68]
Other Infectious disease deaths from *Shigella*-induced stunting as a percentage of total *Shigella* deaths	23	15	20	16	19	16	22	21
	[15; 29]	[10; 20]	[13; 26]	[10; 21]	[12; 24]	[11; 21]	[14; 28]	[14; 27]
Total DALYs* (1000 s)	6,366.9	89.6	3,925.1	0.6	1,456.7	6.2	7,797.4	11,845.1
	[4,800.8; 8,251.8]	[67.2; 117.1]	[2,986.6; 5,032.0]	[0.4; 0.8]	[1,075.0; 1,937.5]	[4.7; 8.0]	[5,906.3; 10,049.4]	[8,928.9; 15,346.9]
Total DALYS* /100,000 children**	336.6	139.3	621.7	0.9	87.0	2.8	364.6	260.0
	[253.8; 436.2]	[104.5; 182.1]	[473.1; 797.0]	[0.6; 1.2]	[64.2; 115.7]	[2.1; 3.6]	[276.2; 469.9]	[196.0; 336.9]
Outpatient treatment costs (millions US$)	212.9	12.8	220.2	0.2	189.7	1.1	324.3	636.9
	[147.4; 279.8]	[8.9; 16.8]	[152.4; 289.3]	[0.2; 0.3]	[131.3; 249.3]	[0.7; 1.4]	[224.6; 426.2]	[441.0; 837.0]
Inpatient treatment costs (millions US$)	178.6	15.5	215.8	0.3	225.3	1.2	250.4	636.7
	[97.8; 277.7]	[8.5; 24.1]	[118.2; 335.6]	[0.2; 0.5]	[123.4; 350.3]	[0.7; 1.9]	[137.2; 389.4]	[348.7; 990.1]

*SHIGELLA vaccination IMPACT*								
MSD episodes averted (millions)	24.97	1.24	19.62	0.02	14.95	0.11	35.85	60.90
	[18.57; 31.45]	[0.92; 1.56]	[14.59; 24.71]	[0.01; 0.02]	[11.11; 18.82]	[0.08; 0.13]	[26.65; 45.15]	[45.28; 76.71]
*Shigella*-induced stunting cases averted (millions)	4.30	0.19	3.32	0.002	3.03	0.02	6.39	10.86
	[2.46; 6.56]	[0.11; 0.29]	[1.90; 5.06]	[0.001; 0.004]	[1.7; 4.6]	[0.01; 0.02]	[3.65; 9.75]	[6.20; 16.56]
Direct diarrheal deaths averted (1000 s)	64.84	1.04	42.44	0.007	18.16	0.08	85.37	126.57
	[43.27; 90.95]	[0.69; 1.48]	[28.40; 59.25]	[0.005; 0.011]	[11.93; 26.12]	[0.05; 0.11]	[57.03; 119.57]	[84.41; 177.84]
Direct diarrheal deaths averted/100,000 FVC	4.15	1.79	7.80	0.01	1.20	0.04	4.54	3.21
	[2.77; 5.82]	[1.19; 2.55]	[5.22; 10.89]	[0.01; 0.02]	[0.79; 1.72]	[0.02; 0.05]	[3.03; 6.35]	[2.14; 4.51]
Infectious disease deaths due to induced stunting averted (1000 s)	19.15	0.18	10.72	0.001	4.16	0.01	23.48	34.23
	[10.25; 31.07]	[0.10; 0.30]	[5.75; 17.31]	[0.001; 0.002]	[2.19; 6.87]	[0.01; 0.02]	[12.59; 38.01]	[18.31; 55.54]
Infectious disease deaths due to induced stunting/100,000 FVC	1.23	0.32	1.97	0.003	0.27	0.007	1.25	0.87
	[0.66; 1.99]	[0.17; 0.52]	[1.06; 3.18]	[0.001; 0.004]	[0.14; 0.45]	[0.004; 0.012]	[0.67; 2.02]	[0.46; 1.41]
Total deaths averted* (1000 s)	83.99	1.22	53.16	0.009	22.32	0.09	108.85	160.80
	[55.51; 119.06]	[0.81; 1.75]	[35.32; 74.90]	[0.006; 0.013]	[14.59; 32.28]	[0.06; 0.13]	[72.19; 154.03]	[106.38; 228.16]
Total deaths averted*/100,000 FVC***	5.38	2.11	9.77	0.02	1.47	0.04	5.78	4.08
	[3.55; 7.62]	[1.39; 3.02]	[6.49; 13.76]	[0.01; 0.02]	[0.96; 2.13]	[0.029; 0.062]	[3.84; 8.18]	[2.70; 5.78]
Total DALYs* averted (1000 s)	2,779.5	41.1	1,782.4	0.3	744.4	3.0	3,625.1	5,350.7
	[1,843.0; 3,933.7]	[27.1; 58.7]	[1,186.1; 2,510.8]	[0.2; 0.4]	[487.1; 1,073.3]	[2.0; 4.3]	[2,408.5; 5,117.7]	[3,554.8; 7,580.2]
Total DALYs* averted/100,000 FVC***	177.9	70.9	327.5	0.5	49.0	1.5	192.6	135.7
	[118.0; 251.8]	[46.8; 101.3]	[218.0; 461.4]	[0.4; 0.8]	[32.1; 70.7]	[1.0; 2.1]	[128.0; 271.9]	[90.1; 192.2]
Vaccination costs (millions US$)	5,685	206	1,928	191	5,365	730	6,835	14,104
	[4,509; 9,190]	[163; 335]	[1,523; 3,144]	[151; 313]	[4,239; 8,760]	[576; 1,192]	[5,421; 11,057]	[11,165; 22,933]
Vaccination costs/100,000 FVC***	363,978	355,339	354,231	352,802	353,201	352,645	363,155	357,608
	[288,677; 588,335]	[280,891; 578,924]	[279,949; 577,793]	[278,730; 576,355]	[279,075; 576,739]	[278,583; 576,204]	[288,028; 587,469]	[283,072; 581,456]
Administration costs/100,000 FVC***	105,908	98,125	97,127	95,840	96,199	95,698	105,166	100,169
	[73,377; 138,875]	[67,984; 128,669]	[67,293; 127,360]	[66,401; 125,672]	[66,650; 126,144]	[66,303; 125,487]	[72,863; 137,902]	[69,401; 131,350]
Medical costs averted (millions US$)	191.8	15.7	226.2	0.2	225.4	1.3	297.6	660.6
	[115.0; 288.0]	[9.2; 23.9]	[134.4; 341.4]	[0.1; 0.4]	[132.5; 342.1]	[0.7; 1.9]	[179.3; 446.3]	[392.2; 997.1]
Net costs (millions US$)	5,493	190	1,701	191	5,139	728	6,537	13,444
	[4,323; 9,000]	[147; 320]	[1,286; 2,922]	[151; 312]	[4,007; 8,535]	[575; 1,191]	[5,124; 10,759]	[10,496; 22,277]
ICER without stunting burden (2016 US$ / DALY)	2,485	5,339	1,175	746,611	8,282	276,909	2,245	3,114
	[1,700; 4,949]	[3,562; 11,042]	[779; 2,450]	[504,555; 1,505,798]	[5,561; 16,963]	[190,741; 549,697]	[1,532; 4,492]	[2,117; 6,276]
ICER with stunting burden (2016 US$ / DALY)	1,976	4,627	955	649,033	6,904	239,964	1,803	2,513
	[1,350; 3,950]	[3,093; 9,584]	[631; 1,999]	[441,268; 1,314,833]	[4,647; 14,221]	[165,492; 477,401]	[1,229; 3,621]	[1,708; 5,088]

*Note:* Though vaccinations occur annually from 2025 to 2034, impacts are projected over the first five years of the vaccinated child’s life. Thus, the last year included in impact estimates is 2039.

* Total deaths and DALYS are the sum of *Shigella* burden attributed to diarrhea from *Shigella* infection (direct) and *Shigella*-induced deaths from other infectious diseases.

** Children: children under 5 years of age.

*** Fully vaccinated children (FVC): Number of eligible children who received all three doses of the vaccine.

Introducing ETEC or *Shigella* vaccines would prevent 92,000 [61,200; 129,900] ETEC and 126,600 [84,300; 179,600] *Shigella* direct deaths and 21,800 [11.29; 34.75] ETEC- and 34,200 [18,000; 56,000] *Shigella*-induced stunting deaths from other infectious diseases over the first 10 years (Table 3, Table 4). ETEC and *Shigella* vaccination would prevent 17.7 [10.0; 27.0] million cases of moderate or severe stunting. ETEC vaccination would prevent 2.9 [1.9; 4.1] deaths/100,000 vaccinated children, globally, with the greatest benefit in AFRO at 4.7 [3.1; 6.7] deaths averted/100,000 vaccinated children. *Shigella* vaccination would prevent 4.0 [2.7; 5.9] deaths/100,000 vaccinated children, globally, with the highest reduction in EMRO at 9.8 [6.5; 13.9] deaths averted/100,000 vaccinated children. Vaccination would prevent 51% and 52% of the global ETEC and *Shigella* diarrheal episodes, respectively.

We also analyzed results for Gavi-eligible countries only. These 48 countries account for over 60% of the total ETEC and *Shigella* burden (Table 3, Table 4). ETEC and *Shigella* vaccines in these countries can prevent over 66,700 [44,100; 94,700] ETEC and 108,000 [72,200; 156,000] *Shigella* deaths. The vaccines are projected to avert 61.4 [44.6; 77.4] million cases of MSD and approximately 10 [6; 15] million cases of moderate-to-severe stunting over the first 5 years of life in GAVI-eligible LMICs.

### 3.2 *Cost-effectiveness of ETEC and* Shigella *vaccination*

Globally, the ICER for ETEC vaccination is estimated at $3508 [2357; 7037]/DALY averted and $2513 [1708; 5088]/DALY for *Shigella* vaccination from 2025 to 2034 (Table 3, Table 4). Regional ETEC ICERs range from $2177 [1469; 4334]/DALY in AFRO to $19,434 [12,789; 39,598]/DALY in EURO. Regional *Shigella* ICERs range from $955 [631; 1999]/DALY in EMRO to $649,033 [441,268; 1,314,833]/DALY in EURO.

**Fig. 1:**
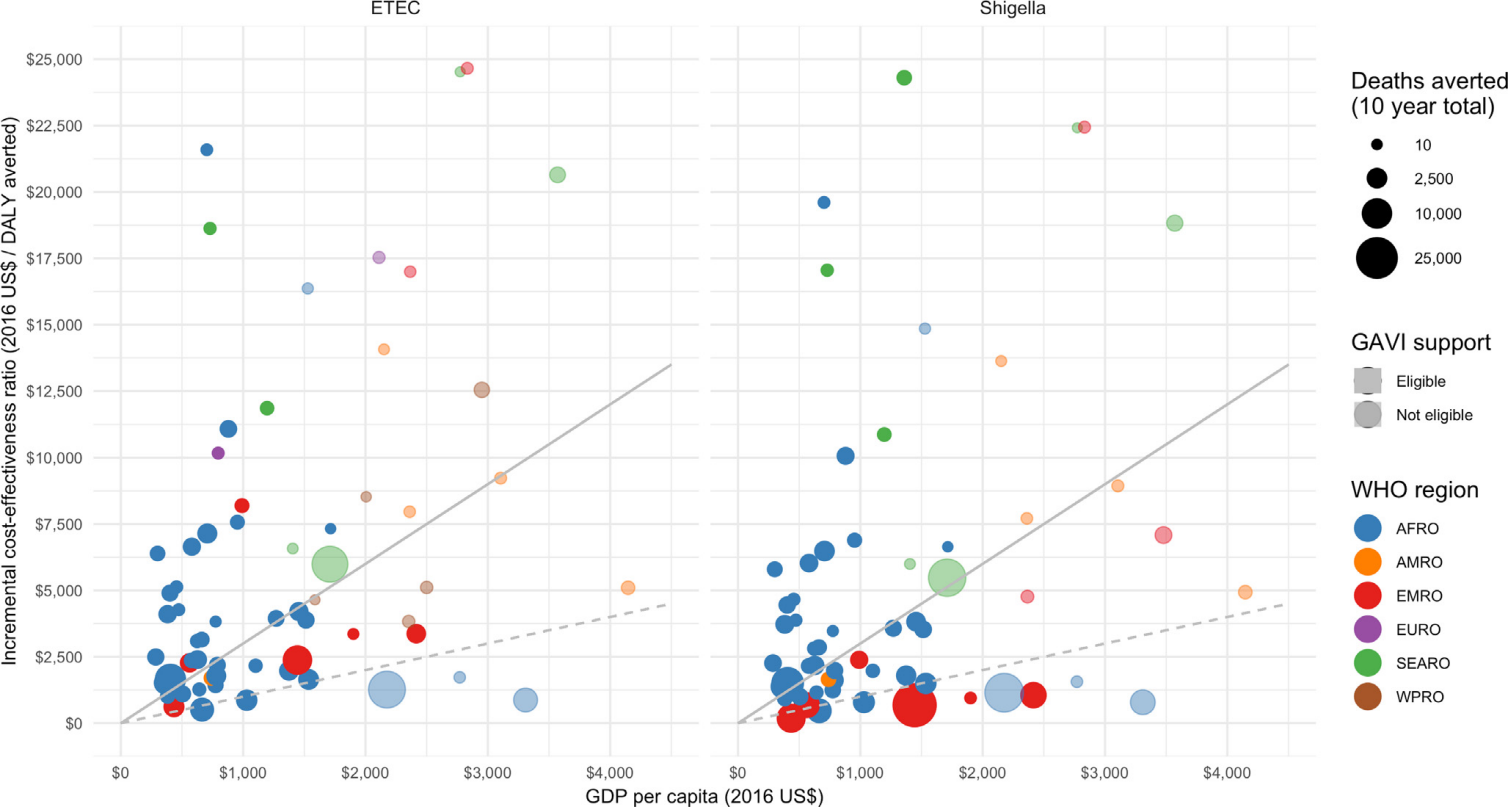
Incremental cost-effectiveness of ETEC and *Shigella* standalone vaccines by national Gross Domestic Product (2025–2034). The solid line represents a threshold of ICERs relative to values of three times the GDP, while the dashed line represents ICERS relative to GDP. Countries following below either line would be considered cost-effective based on each criterion.

ETEC and *Shigella* vaccines met the ‘cost-effective’ threshold (ICER < 3XGDP) in 27 [10; 34] and 29 [14; 37] countries, respectively (Table 5), and ‘very cost-effective’ threshold (ICER < GDP) in 6 [1; 8] and 11 [3; 14] countries, respectively (Table 6). The majority of these countries are in AFRO (Fig. 1). When only countries with ‘cost-effective’ ICERs are considered, the global ETEC ICER is $1632 [1449; 2530]/DALY and *Shigella* is $1117 [1045; 1687]/DALY, lower than the global ICERs when all countries are considered. In countries meeting the ‘very cost-effective’ threshold, global ICERs are most favorable at $1061 [828; 1941]/DALY and $810 [540; 1413]/DALY for ETEC and *Shigella* vaccination, respectively. Implementing in the ‘cost-effective’ countries would achieve 53% and 66% of the potential benefit ETEC and *Shigella* vaccination, respectively, in all countries, at 25% and 30% of the net costs.

**Table 5:** Cost-effectiveness of standalone vaccines for ETEC and *Shigella*, when limited to settings with an ICER less than three times the GDP threshold, by region, 2025–2034. Results from uncertainty analysis are listed below model estimates. Upper and Lower represent 95% uncertainty intervals for each estimate.

	AFRO	AMRO	EMRO	SEARO	WPRO**	GAVI-eligible	Global
*ETEC vaccination*							
Number of countries ICER < 3 X GDP	17	3	4	–	3	19	27
	[7; 21]	[1; 4]	[2; 5]	–	[ND; 3]	[6; 24]	[10; 34]
Total deaths* averted (1000 s)	45.6	1.0	13.6	–	0.4	35.0	60.7
	[23.6; 71.3]	[0.2; 1.5]	[2.7; 19.4]	–	[ND; 0.6]	[10.0; 59.3]	[26.8; 116.1]
Total deaths* averted/100,000 FVC***	7.2	2.5	4.6	–	2.2	5.4	6.1
	[5.5; 9.9]	[1.2; 3.7]	[2.8; 6.2]	–	[ND; 3.0]	[4.1; 7.6]	[3.9; 8.0]
Total DALYs* averted (1000 s)	1,561	35	464	–	13	1,194	2,073
	[807; 2,440]	[8; 51]	[93; 659]	–	[ND; 19]	[341; 2,026]	[915; 3,970]
Total DALYs* averted/100,000 FVC***	246	86	155	–	76	185	209
	[190; 340]	[40; 128]	[94; 211]	–	[ND; 100]	[140; 260]	[133; 273]
Vaccination costs (millions US$)	2,259	146	1,055	–	60	2,295	3,520
	[1,550; 2,944]	[69; 183]	[332; 1,631]	–	[ND; 95]	[821; 3,026]	[2,333; 7,928]
Vaccination costs/100,000 FVC***	355,702	356,373	353,238	–	352,397	356,570	354,931
	[274,110; 561,392]	[265,267; 557,867]	[253,764; 557,408]	–	[ND; 546,333]	[274,842; 563,324]	[272,039; 561,229]
Administration costs/100,000 FVC***	98,453	99,057	96,233	–	95,475	99,234	97,757
	[65,886; 125,759]	[63,968; 126,238]	[60,760; 123,783]	–	[ND; 122,304]	[66,462; 127,364]	[65,076; 124,893]
Medical costs averted (millions US$)	82	10	43	–	2	80	137
	[34; 127]	[3; 17]	[7; 62]	–	[ND; 4]	[13; 125]	[49; 374]
ICER (2016 US$/DALY)	1,395	3,871	2,181	–	4,474	1,856	1,632
	[982; 2,154]	[2,479; 9,664]	[1,203; 3,681]	–	[ND; 6,655]	[1,206; 2,762]	[1,449; 2,530]

*Shigella vaccination*							
Number of countries ICER < 3 X GDP	18	3	8	–	–	22	29
	[8; 23]	[1; 4]	[5; 8]	–	–	[10; 27]	[14; 37]
Total deaths* averted (1000 s)	52.7	1.1	53.0	–	–	76.5	106.8
	[27.1; 91.0]	[0.2; 1.6]	[31.9; 71.9]	–	–	[41.1; 121.4]	[59.4; 188.3]
Total deaths* averted/100,000 FVC***	7.9	2.6	10.7	–	–	10.1	8.8
	[6.1; 10.9]	[1.2; 3.9]	[8.1; 15.2]	–	–	[8.2; 14.2]	[5.3; 11.9]
Total DALYs* averted (1000 s)	1,746	36	1,777	–	–	2,556	3,559
	[901; 3,010]	[8; 53]	[1,070; 2,412]	–	–	[1,377; 4,041]	[1,983; 6,255]
Total DALYs* averted/100,000 FVC***	260	89	357	–	–	339	294
	[202; 361]	[41; 130]	[270; 508]	–	–	[273; 476]	[176; 395]
Vaccination costs (millions US$)	2,386	146	1,763	–	–	2,693	4,296
	[1,650; 3,237]	[71; 187]	[1,250; 2,426]	–	–	[1,811; 3,597]	[3,030; 9,202]
Vaccination costs/100,000 FVC***	355,503	356,373	354,360	–	–	356,897	355,062
	[275,835; 560,639]	[268,790; 558,302]	[272,641; 560,870]	–	–	[276,720; 562,627]	[273,263; 560,780]
Administration costs/100,000 FVC***	98,273	99,057	97,243	–	–	99,529	97,876
	[66,166; 125,794]	[64,733; 125,497]	[65,110; 124,785]	–	–	[67,012; 127,662]	[65,318; 124,896]
Medical costs averted (millions US$)	98	10	213	–	–	184	321
	[38; 151]	[3; 17]	[74; 302]	–	–	[81; 269]	[116; 652]
ICER (2016 US$/DALY)	1,311	3,750	872	–	–	981	1,117
	[899; 1,960]	[2,465; 9,416]	[539; 1,260]	–	–	[713; 1,366]	[1,045; 1,687]

* Total deaths and DALYS are the sum of burden attributed to diarrhea from ETEC or Shigella infection and ETEC- or Shigella-induced deaths from other infectious diseases.

** “ND” indicates ‘not defined’ as there were no WPRO countries that met the threshold under the predicted lower bounds (2.5%) of the 95% uncertainty intervals.

*** Fully vaccinated children (FVC): Number of eligible children who received all three doses of the vaccine.

**Table 6:** Cost-effectiveness of standalone vaccines for ETEC and *Shigella*, when limited to settings with an ICER less than GDP threshold, by region, 2025–2034. Results from uncertainty analysis are listed below model estimates. Upper and Lower represent 95% uncertainty intervals for each estimate.

	AFRO	AMRO	EMRO	SEARO	WPRO	GAVI-eligible**	Global
*ETEC vaccination*							
Number of countries ICER < GDP	6	–	–	–	–	3	6
	[1; 7]	–	–	–	–	[ND; 4]	[1; 8]
Total deaths* averted (1000 s)	32	–	–	–	–	8	32
	[3; 46]	–	–	–	–	[ND; 14]	[3; 48]
Total deaths* averted/100,000 FVC**	9	–	–	–	–	16	5
	[6; 12]	–	–	–	–	[ND; 22]	[1; 7]
Total DALYs* averted (1000 s)	1108.1	–	–	–	–	259.4	1108.1
	[119; 1590]	–	–	–	–	[ND; 477]	[119; 1645]
Total DALYs* averted/100,000 FVC**	320	–	–	–	–	548	320
	[196; 416]	–	–	–	–	[ND; 756]	[196; 407]
Vaccination costs (millions US$)	1226	–	–	–	–	175	1226
	[229; 1690]	–	–	–	–	[ND; 387]	[229; 1732]
Vaccination costs/100,000 FVC***	354,236	–	–	–	–	369,582	354,236
	[272,778; 559,003]	–	–	–	–	[ND; 500,700]	[272,788; 559,003]
Administration costs/100,000 FVC***	97,132	–	–	–	–	110,957	97,132
	[64,858; 122,847]	–	–	–	–	[ND; 136,183]	[64,853; 122,847]
Medical costs averted (millions US$)	50	–	–	–	–	4	50
	[14; 75]	–	–	–	–	[ND; 13]	[14; 81]
ICER (2016 US$/DALY)	1,061	–	–	–	–	660	1,061
	[746; 1,941]	–	–	–	–	[ND; 845]	[828; 1,941]

*Shigella vaccination*							
Number of countries ICER < GDP	7	–	4	–	–	8	11
	[1; 8]	–	[2; 5]	–	–	[2; 10]	[3; 14]
Total deaths* averted (1000 s)	40	–	44	–	–	55	84
	[4; 55]	–	[11; 65]	–	–	[11; 82]	[15; 120]
Total deaths* averted/100,000 FVC**	10	–	15	–	–	14	12
	[6; 14]	–	[10; 20]	–	–	[10; 19]	[8; 16]
Total DALYs* averted (1000 s)	1312	–	1476	–	–	1856	2787
	[138; 1806]	–	[365; 2181]	–	–	[365; 2,727]	[510; 3,996]
Total DALYs* averted/100,000 FVC**	338	–	494	–	–	478	406
	[213; 453]	–	[320; 666]	–	–	[323; 647]	[267; 542]
Vaccination costs (millions US$)	1,375	–	1,055	–	–	1,379	2,430
	[256; 1,759]	–	[442; 1,644]	–	–	[442; 1,778]	[713; 3,353]
Vaccination costs/100,000 FVC***	354,016	–	353,238	–	–	355,120	353,678
	[271,616; 556,691]	–	[264,699; 556,090]	–	–	[266,212; 556,324]	[271,471; 557,353]
Administration costs/100,000 FVC***	96,933	–	96,233	–	–	97,928	96,629
	[64,380; 123,469]	–	[63,434; 123,544]	–	–	[64,354; 125,143]	[64,302; 123,565]
Medical costs averted (millions US$)	60	–	113	–	–	123	173
	[16; 85]	–	[13; 168]	–	–	[13; 178]	[31; 256]
ICER (2016 US$/DALY)	1,002	–	638	–	–	677	810
	[674; 1,865]	–	[402; 1,187]	–	–	[433; 1,187]	[540; 1,413]

* Total deaths and DALYS are the sum of burden attributed to diarrhea from ETEC or Shigella infection and ETEC- or Shigella-induced deaths from other infectious diseases.

** “ND” indicates ‘not defined’ as there were no GAVI-eligible countries that met the threshold under the predicted lower bounds (2.5%) of the 95% uncertainty intervals.

*** Fully vaccinated children (FVC): Number of eligible children who received all three doses of the vaccine

ICERs for ETEC and *Shigella* vaccines were more favorable at $2909 [1964; 5788]/DALY and $1803 [1229; 3621]/DALY when only considering the 48 GAVI-eligible countries (Table 3, Table 4). In GAVI-eligible countries where vaccines were considered ‘cost-effective’ (ICER < 3XGDP, ETEC: 19 [6; 24], *Shigella*; 22 [10; 27] countries), ICERs were $1856 [1206; 2762]/DALY and $981 [713; 1366]/DALY for ETEC and *Shigella* vaccines, respectively (Table 5). ICERs improved further to $660 [not defined; 845]/DALY (ETEC) and $677 [433; 1187]/DALY (*Shigella*) when including only ‘very cost-effective’ (ICER < GDP, ETEC: four countries, *Shigella*; nine countries) GAVI-eligible countries (Table 6).

### 3.3 Sensitivity and uncertainty

**Fig. 2:**
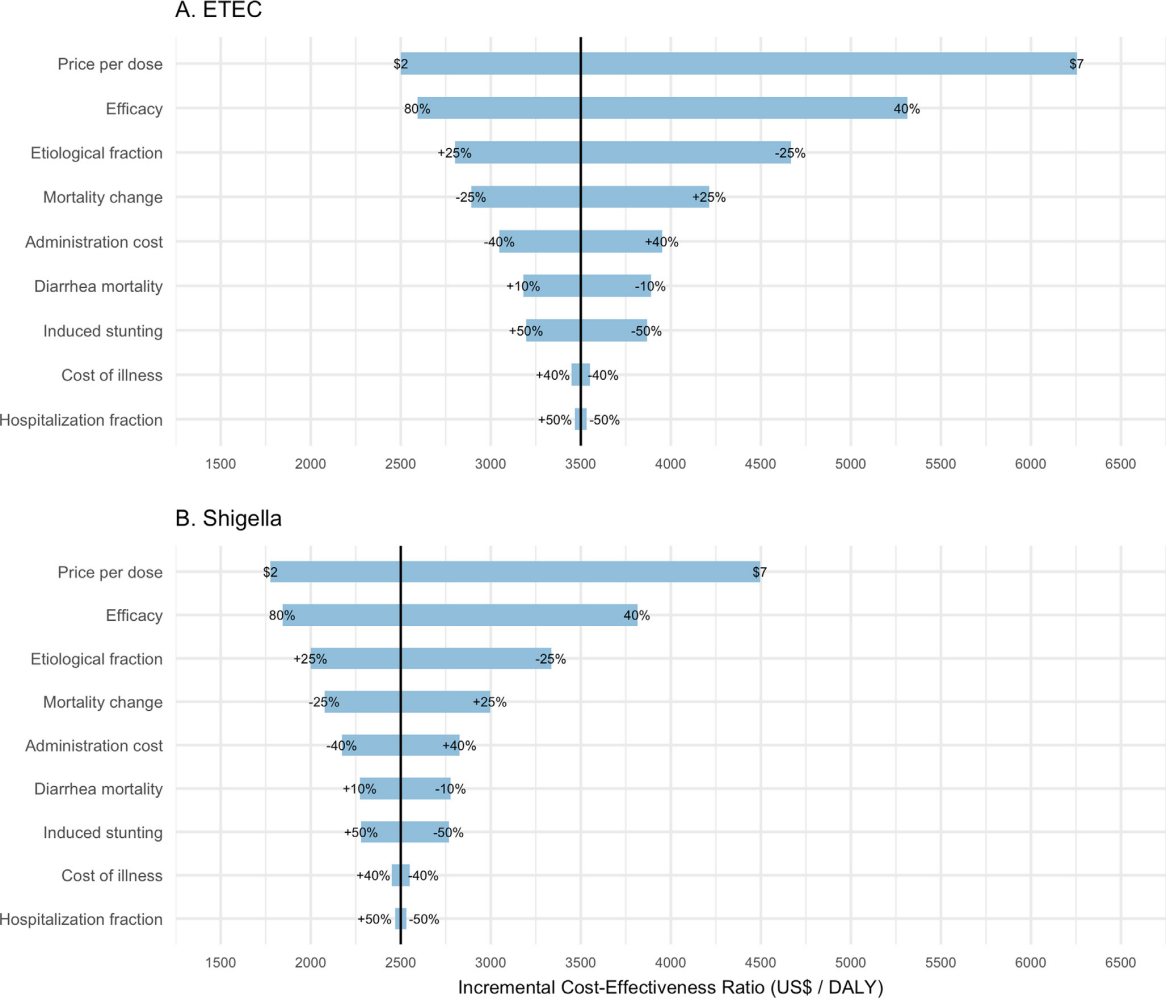
Tornado diagram showing results of one-way PSA exploring the affect key input variables have on cost-effectiveness of ETEC and *Shigella* standalone vaccines, in 79 LMICs from 2025 to 2034. Ranges of variables (listed in Table 2) are displayed at the end of the corresponding bar. Price per dose is varied by $US ranging from a low estimate of $2/dose to a high estimate of $7/dose. ‘Etiological fraction’ is variation in the fraction of overall diarrheal mortality attributed to ETEC and *Shigella* diarrhea. ‘Mortality change’ is variation in the rates diarrheal mortality projected in years 2025–2034. ‘Induced stunting’ refers to the number of other infectious disease deaths caused by ETEC or *Shigella* induced stunting. ‘Hospitalized fraction’ is variation in the fraction of children hospitalized (1 in 8 referred to inpatient facility).

Globally, the most influential variables on ICER estimates were vaccine price per dose (range; $3765/DALY [ETEC] and $2727/DALY [*Shigella*]) and efficacy (range; $2729/DALY [ETEC] and $1977/DALY [*Shigella*]), followed by the etiological fraction attributed to each pathogen (range; $1871/DALY [ETEC] and $1340/DALY [*Shigella*]) (Fig. 2A and B). Variation in projected mortality change (range; $1326/DALY [ETEC] and $923/DALY [*Shigella*]) was also influential on ICERs.

## 4 Discussion

Our analysis is the first evaluating the impact and cost-effectiveness of potential ETEC and *Shigella* vaccine candidates in children in low and lower-middle income countries. The impact of vaccination on stunting and deaths from other infectious diseases makes ETEC and *Shigella* vaccination more compelling and cost-effective. These vaccines could avert over 274,000 ETEC and *Shigella* attributable deaths in the first decade, a 47% reduction in mortality. Including benefits of stunting averted results in much lower ICERs and increases the number of countries where vaccination is cost-effective.

Our results suggest that there is heterogeneity in vaccine impact and cost-effectiveness across regions and by Gavi eligibility. While the majority of the averted burden for both ETEC and *Shigella* vaccines is in AFRO, substantial burden is also averted in EMRO. Introducing vaccines only in high burden countries and/regions could reduce cost without substantial reductions in health impact. This is clearest when calculating impact for those countries where vaccines are cost-effective. The number of deaths averted per 100,000 fully vaccinated children for an ETEC vaccine increases, while still averting the majority of preventable total ETEC deaths. Gavi-eligible countries may also benefit greatly as these countries experience a large share of projected disease burden. Vaccine introduction in these countries alone could have a substantial impact on burden. It will be important for country policy makers to understand disease heterogeneity when evaluating whether or not to introduce these vaccines.

There is evidence that childhood stunting may be associated with chronic diseases such as heightened prevalence of high blood pressure, impaired fasting glucose, and increased body mass index [9]. If ETEC and *Shigella* infections increase chronic disease through induced stunting, vaccination could be even more impactful and cost-effective than initially realized. In addition to chronic disease risk, early childhood stunting has been shown to be associated with decreased earnings and fewer completed years of school [51]. If ETEC and *Shigella* vaccination could reduce stunting and these long-term health and development consequences, then vaccination is more economically viable than indicated in the results from this study.

The model incorporates projections based on population forecasts and past diarrheal mortality trends that could contribute to underestimation of vaccine impacts. First, while the past two decades have seen consistent declines in diarrheal mortality [52], additional factors may alter this trend. Global urbanization rates are increasing in low- and lower-middle income countries, placing larger populations at risk as growth overwhelms infrastructure, forcing many into living in underserved informal settlements. Risk for infectious diseases increase in these environments due to overcrowding with lack of safe sanitation and clean water [53]. While moving to urban areas could increase access to health care and therefore reduce mortality, this is highly dependent on individual household economic status and their community’s urban infrastructure which is highly variable within and between cities. There is some evidence that a move to urban areas reduces access to health care [54]. Second, increasing antibiotic resistance and climate change may reverse gains made over the past few years by increasing risk of exposure, severe disease, and death. Third, vaccination impact could be underestimated if these vaccines induce herd protection. Fourth, dmLT adjuvant is in current formulation which has potential to improve protection. Evidence of mucosal immune responses induced by ETVAX vaccine may provide some protection against ETEC colonization factor antigens not included in the vaccine [55].

There are several limitations to this analysis. First, there are no vaccine trial-derived measures of vaccine efficacy and duration. As these vaccine candidates are under development, we do not know their price, thus our cost-effectiveness estimates may be higher or lower than projected. Furthermore, our cost-effectiveness estimates are dependent on assuming that these two vaccines would have the same price, efficacy and coverage.

No treatment-seeking or hospitalization rates exist for children experiencing ETEC and *Shigella* episodes in most of our study countries. We used DHS data on treatment seeking for diarrhea and assumed a proportion of those children are hospitalized. We also adjusted our fraction of MSD episodes based on GEMS methodology. Thus, we have assumed that all MSD cases access treatment which may underestimate the true number of child MSD. We modelled cost of illness based on available estimates—this approach may over- or under-estimate the medical cost averted due to vaccination in many countries. For most input parameters, we have limited information on the true degree of uncertainty and thus we rely on simple distribution types and measures of dispersion. We did not estimate waning in our model or the impact of partial protection from receiving less than three doses.

A final limitation is the rate of decline for diarrheal mortality. We assumed mortality decline mirrors the decline seen during 2000–2013. However, studies are actively evaluating whether the rate of decline is decreasing. Incorporating these findings may alter our estimates of vaccine impact by increasing the number of deaths averted.

This analysis considered the potential impact and cost-effectiveness of vaccines that are in development. It shows the importance of including the expanded impact of vaccination to prevent stunting and resulting deaths due to other infectious disease. To capture the true value of these vaccines, it is important to quantify the expanded effects of enteric pathogens. Burden was concentrated in a few regions, indicating that introduction in these countries could potentially avert the majority of disease burden globally. As these vaccines undergo further clinical development, it will be important to reassess the potential vaccine impact as data on efficacy, duration of protection, and diarrheal impact on stunting becomes available.

## Conflict of interest

The authors have no conflicts of interest to declare.
