# Supplementary material for: Potential impact and cost-effectiveness of future ETEC and Shigella vaccines in 79 low- and lower middle-income countries
Source: Vaccine X. 2019 Apr 18;2:100024. doi: 10.1016/j.jvacx.2019.100024 (PMC6668229; doi:10.1016/j.jvacx.2019.100024)

**Supplemental Materials**

**Supplement Figure 1:** Projected trends in childhood ETEC and *Shigella* mortality among children under five in 79 LMICs by WHO region, 2020-2050.
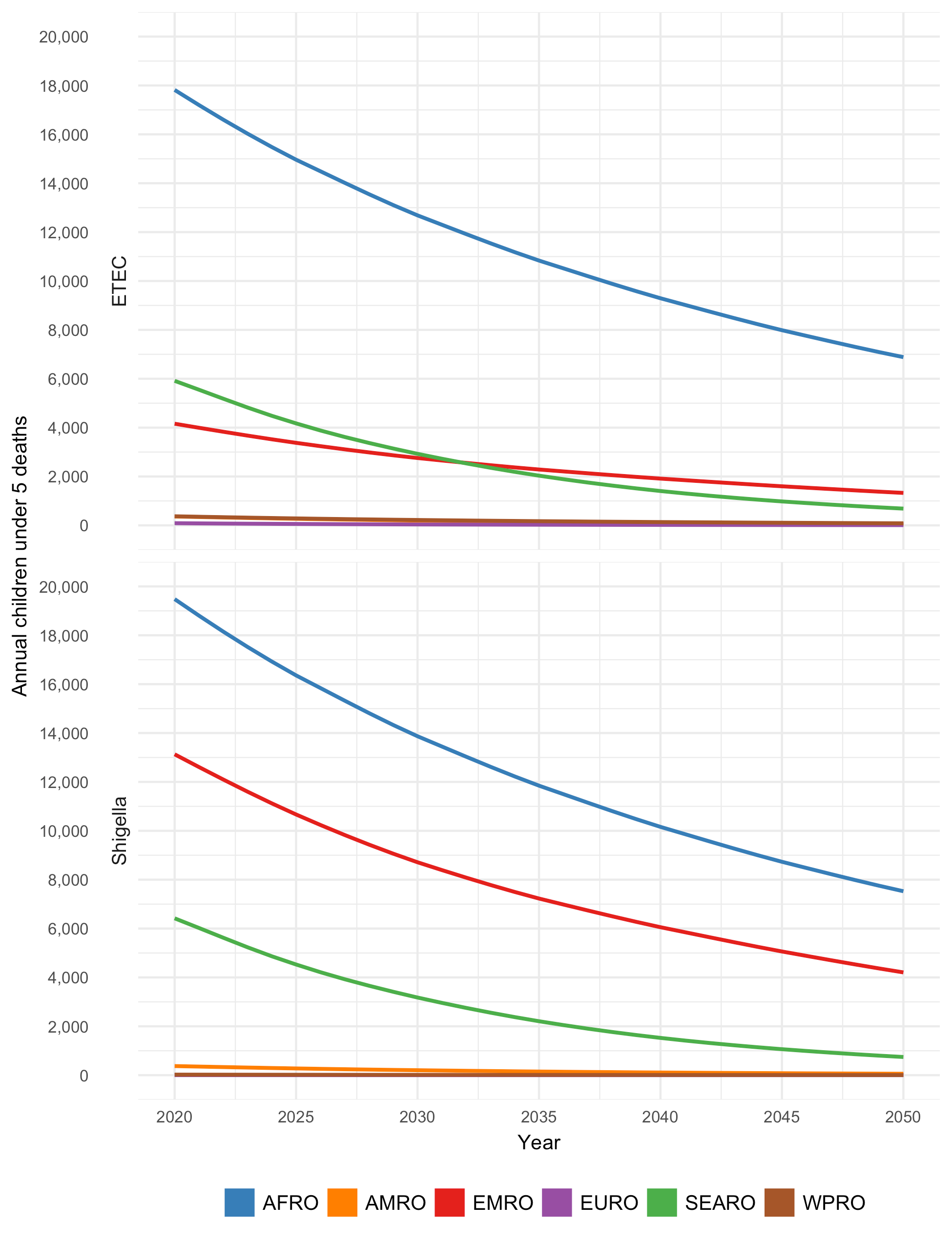


**Supplement Figure 2:** Projected trends in population of children under five in 79 LMICs by WHO region, 2020-2050.


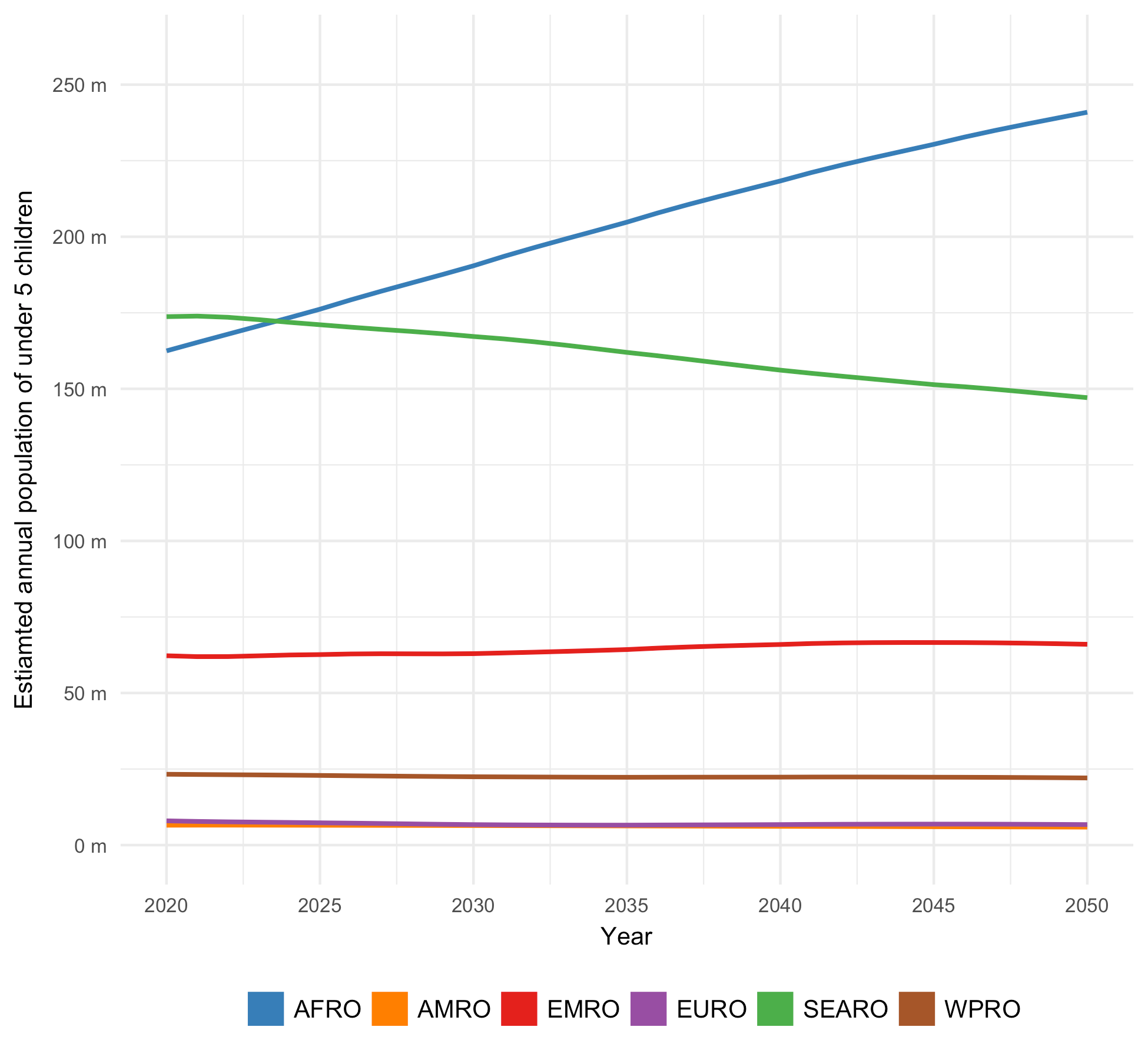

Supplement: Supplementary data 2 [file mmc2.docx]
